# A Generic Transferable EEG Decoder for Online Detection of Error Potential in Target Selection

**DOI:** 10.3389/fnins.2017.00226

**Published:** 2017-05-02

**Authors:** Saugat Bhattacharyya, Amit Konar, D. N. Tibarewala, Mitsuhiro Hayashibe

**Affiliations:** ^1^CAMIN Team, INRIA-LIRMM, University of MontpellierMontpellier, France; ^2^Department of Electronics and Telecommunication Engineering, Jadavpur UniveristyKolkata, India; ^3^School of Bioscience and Engineering, Jadavpur UniveristyKolkata, India

**Keywords:** transfer learning, error related potential, ensemble classifier, electroencephalography, brain-computer interface

## Abstract

Reliable detection of error from electroencephalography (EEG) signals as feedback while performing a discrete target selection task across sessions and subjects has a huge scope in real-time rehabilitative application of Brain-computer Interfacing (BCI). Error Related Potentials (ErrP) are EEG signals which occur when the participant observes an erroneous feedback from the system. ErrP holds significance in such closed-loop system, as BCI is prone to error and we need an effective method of systematic error detection as feedback for correction. In this paper, we have proposed a novel scheme for online detection of error feedback directly from the EEG signal in a transferable environment (i.e., across sessions and across subjects). For this purpose, we have used a P300-speller dataset available on a BCI competition website. The task involves the subject to select a letter of a word which is followed by a feedback period. The feedback period displays the letter selected and, if the selection is wrong, the subject perceives it by the generation of ErrP signal. Our proposed system is designed to detect ErrP present in the EEG from new independent datasets, not involved in its training. Thus, the decoder is trained using EEG features of 16 subjects for single-trial classification and tested on 10 independent subjects. The decoder designed for this task is an ensemble of linear discriminant analysis, quadratic discriminant analysis, and logistic regression classifier. The performance of the decoder is evaluated using accuracy, F1-score, and Area Under the Curve metric and the results obtained is 73.97, 83.53, and 73.18%, respectively.

## 1. Introduction

Technological advances over the last couple of decades has led to a rapid advancement in the field of neuroscience such that it is now possible to design and develop flexible and adaptive brain-based technologies to improve brain-computer interactions (Nicolas-Alonso and Gomez-Gil, 2012). Brain-computer interfaces (BCI) aim at providing a direct communication pathway between the human brain and some external devices, like a robotic arm or prosthesis (Millán et al., 2004; Dornhege, 2007; Alwaisiti et al., 2010; Chae et al., 2012).

These BCI technologies follow the principle that the intent of any action and its subsequent planning originates from the brain, which can be extracted, decoded, and analyzed by the use of various brain measures like Electroencephalography (EEG), functional Magnetic Resonance Imaging (fMRI), functional Near Infra Red Spectroscopy (fNIRS), and intra-cortical electrodes (Mason et al., 2007; Schalk, 2009). EEG is the most preferred brain measure among researchers because of its non-invasiveness, portability, easy, and inexpensive availability, and high temporal resolution (Dornhege, 2007; Millán et al., 2010).

EEG based BCI (EEG-BCI) technologies decode the brain signals recorded from the scalp of the electrodes to discriminate among the various intentions of the subject. Based on the cognitive task performed by the subject, various signal modalities can be extracted from the EEG signals. Steady-state visually evoked potential (SSVEP) (Müller-Putz et al., 2006), Slow cortical potential (SCP) (Hinterberger et al., 2004), P300 (Bhattacharyya et al., 2014), Event related desynchronization/synchronization (ERD/ERS) (Bhattacharyya et al., 2014) are the commonly used modalities in BCI research. Till date, most of the current BCI modalities are implemented practically for discrete target selection task.

Recently, a new form of BCI modality, known as *Error Related Potential* (ErrP) (Schalk et al., 2000; Combaz et al., 2012) is gaining a lot of attention among researchers. ErrP signals indicate awareness of the subject toward an occurrence of error. Compared to other BCI modalities, ErrP signals have not yet been widely studied among researchers. Most of the BCI studies are concerned with developing new and efficient algorithms to improve performance of brain-signal classification. Somehow, we would have the situation where the decoder misinterprets the intention of the subject and provides a completely different result. This mainly occurs due to the noisy, non-stationary, non-Gaussian nature of the EEG signal. It is noted, till date, even the most well-trained BCI users have difficulty in reaching an optimal result. To tackle this problem, we require a system to detect errors made by the system or the subject and correct it in subsequent steps. The answer to this problem lies in the human brain itself in the form of ErrP signal.

ErrP signal is usually detected for either of the three cases: (i) when a subject commits errors in a choice reaction task, which is characterized by a negative peak (known as *Error Related Negativity (ERN)*) at around 50–100 ms after the subject's response, followed by a centro-parietal positive peak (denoted as *Pe*) (Falkenstein et al., 2000), (ii) when a person recognizes error in the task performed by a second subject, called *observation ErrP*, and (iii) when a subject observes an agent committing an error, called *interaction ErrP*. For the latter two scenarios, the ErrP usually appears after the presentation of a feedback, which is characterized by positive peak at around 200 ms, followed by a large negative peak at around 250 ms and again a positive deflection at around 320 ms (van Schie et al., 2004; Chavarriaga et al., 2014). The interaction ErrP is more common in discrete target selection tasks in BCI, as such experimental sessions usually includes a feedback.

The ErrP signal is generally used with other signal modalities to detect error in the system. For example, in 2012, Combaz et al. employed ErrP to detect errors in classification of a P300 based mind speller. In 2008, Ferez and Millan employed motor intention to trigger the movement of a cursor left or right, and ErrP is used as a feedback signal to cancel the movement of the cursor, when an error in motor intention is detected. Seno et al. (2010) was one of the first groups to test online automatic error detection from a BCI P300-speller with a specificity of 68% and sensitivity of 62%. Spüler et al. (2012) performed an online study on ErrP detection from 17 normal and 6 motor impaired participants. By including error correction, the normal, and patient participants showed an increase in performance. Researchers in Perrin et al. (2011) tested an automatic error detection system offline and obtained a specificity above 90% and a sensitivity up to 60%. The same group in Perrin et al. (2012), further went on to develop an online error correction system during P300-based spelling task. In this study, the subjects are divided into two groups, high specificity (> 85%) and low specificity (<75%). The high specificity group performed the spelling task much better than the low specificity group and on inclusion of online correction, the average spelling accuracy of the high specificity group increased by 4% from an accuracy of 72% (when no correction was included). Their study did not include transfer learning for cross-subject validation, which is the main objective of our paper.

Reliable classification of mental states while taking into account the change in data distribution between sessions and subjects (termed as *transfer learning*, Samek et al., 2013) has generated a considerable amount of interest among BCI researchers (Kang et al., 2009; Devlaminck et al., 2011; Samek et al., 2013). It allows the classifier to be trained on a fixed set of subjects and test it on a completely different set of subjects. BCI systems till date require a degree of initial subject training which may range from weeks to months before it is fully operational, which often becomes a tedious and time-consuming process and is not practical in designing a real-time BCI. Implementation of a transfer learning framework construction of a cross-subject independent classifier with no prior calibration is a possibility and it has a huge advantage toward real-time implementation of BCI, as it would become more robust and would evolve into a subject-independent zero-training system (Fazli et al., 2009). The main problem that arises from designing such framework is the high inter-subject variability. Due to this issue, a classifier trained with a given number of subjects doesn't perform well for a new independent subject and further degrades its performance. As a result, determination of proper features and generalization of the classifiers is a must to tackle this issue. In the past (Kang et al., 2009; Lotte and Guan, 2010), researchers have averaged the covariance matrix of different subjects toward creating a generalized covariance matrix to improve the cross-subject estimation. Another approach (Devlaminck et al., 2011) toward transfer learning employed common spatial patterns to construct a common feature space among various subjects. A recent study (Samek et al., 2013) employed common spatial patterns and principal component analysis to transfer the non-stationarity within the signal. The method showed some positive result in classifying motor imagery signals. A different approach was taken in Fazli et al. (2009) where the researchers had developed a subject-independent ensemble classifier to detect motor imagery tasks. The ensemble approach provided a robust interpretation of the mental states of participants without any training and only a moderate performance loss. In Waytowich et al. (2016), an unsupervised transfer method, a spectral transfer method using information geometry, was proposed which ranked and combined the unlabeled classes from an ensemble of information geometry classifiers. This method showed that single trial detection is possible using unsupervised transfer learning without a huge training data.

In this paper, we have proposed a subject-independent transferable BCI system to detect error, in the form of ErrP, in a discrete target selection task. An ensemble online decoder (Dietterich, 2000) is constructed using Linear Discriminant Analysis, Quadratic Discriminant Analysis, and Logistic Regression classifiers (Alpaydin, 2004; Hastie et al., 2001) for this purpose. Through this ensemble approach, we aim to stabilize and generalize the performance of the classifier and make it robust in detection of ErrP from EEG signals to be transferable to the ErrP detection in new subject. The complexity of the classifier is kept low to make it more suitable for real-time application. For this purpose, we have employed a competition dataset hosted at Kaggle (BCI Challenge, 2015) based on a P300-speller task and the competition had a similar objective to this paper. One of the top five submission (Barrack, 2015) in the competition used the meta features (trial time stamp, trial session number, etc), means of the EEG for each channel of windows of various lengths and lags and template matching features, which were fed on two regularized support vector machines using linear kernel. This approach scored an area under the curve (AUC) of 76.921% in the leaderboard. The winning solution (Barachant, 2015) produced an AUC score of 84.585% employed features from Xdawn covariances and metadata and classifier using Bagging technique. We have tested the online performance of the BCI system in a simulated real-time environment with the dataset provided.

Rest of the paper is organized as follows: Section II provides information on the experimental setup and the datasets arising from it. Section III discusses on the working principle and methodology of the BCI system. It also gives a detail overview on the construction of the transferable ensemble decoder. The training and online test results are presented in Section IV. Salient features of the work, comparison with state-of-the-art techniques and its future direction are discussed in Section V. Concluding remarks are given in Section VI.

## 2. Datasets and experimental protocol

**Figure 1:**
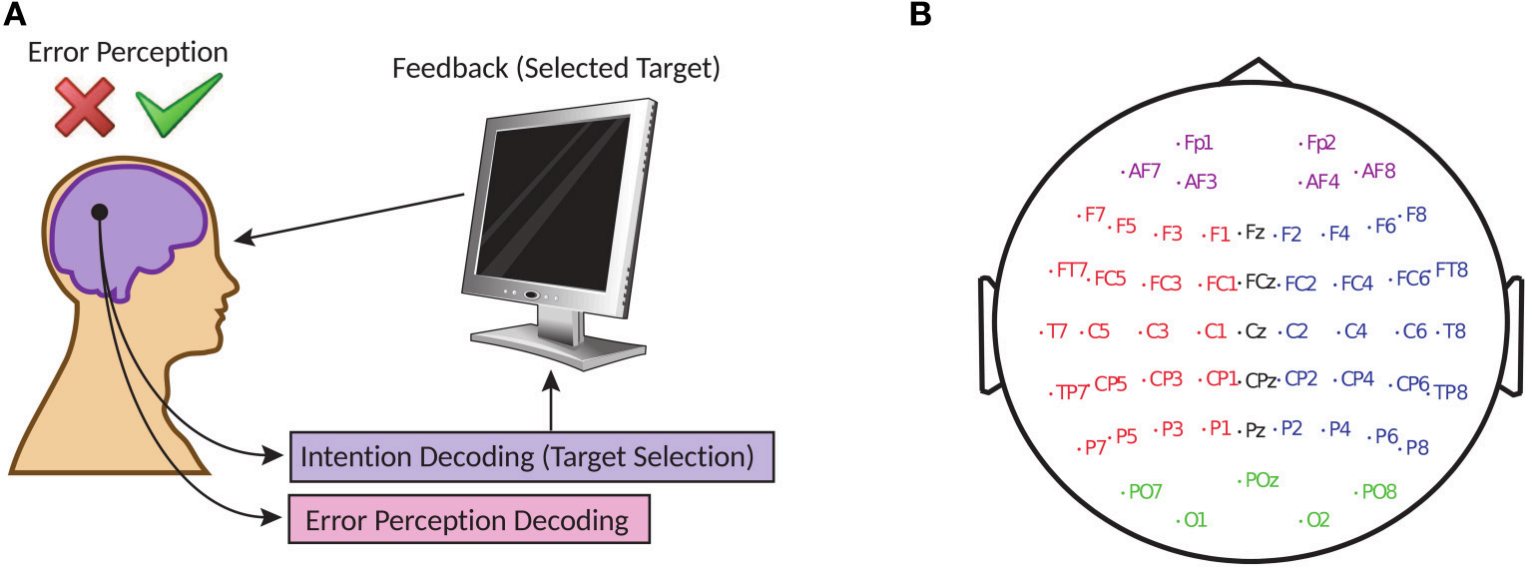
**(A)** Online error potential decoding framework in target selection with BCI **(B)** Electrode locations of the 56 channels arranged in extended 10–20 system.

The dataset for this study is obtained from the “BCI Challenge @ NER 2015” competition hosted at Kaggle (BCI Challenge, 2015). The objective of the competition was to design an error potential detection algorithm capable of detecting erroneous feedback (illustration in Figure 1A) with cross-subject generalization. In this study, we have attempted the same but specifically for online scenario. The EEG dataset contains recording from 26 participants and for this paper, 16 participants are used to train and validate the proposed BCI system, and 10 participants are used for cross-subject testing as new independent group. The subjects in this dataset are in the age range of 20–37 years and none of the subjects had any previous experience with P300-speller paradigm (Farwell and Donchin, 1988) or any other BCI application. Prior to the experiment, the participants signed an informed consent approved by the Local Ethical Committee (BCI Challenge, 2015).

The brain activities of the participants are recorded simultaneously with a 56 channel passive Ag/AgCl EEG sensors (VSM-CTF compatible system) and the placement of the electrodes followed the extended 10–20 system (Figure 1B). The electrodes are referenced to the nose, the ground electrode is placed on the shoulder and the impedances of the electrodes were maintained at 10 kΩ. During acquisition, the signals are sampled at 600 Hz but to aid in its online processing, the signals provided by its contributors (BCI Challenge, 2015) are down-sampled to 200 Hz.

The participants performed a P300-Speller task for this experiment and its in-depth explanation are provided in Perrin et al. (2012). In this paper, we provide a brief description of the task. A standard 6 × 6 matrix of items (alphabets) arranged in a random fashion was used as in Perrin et al. (2012) to design the visual stimuli.

**Figure 2:**
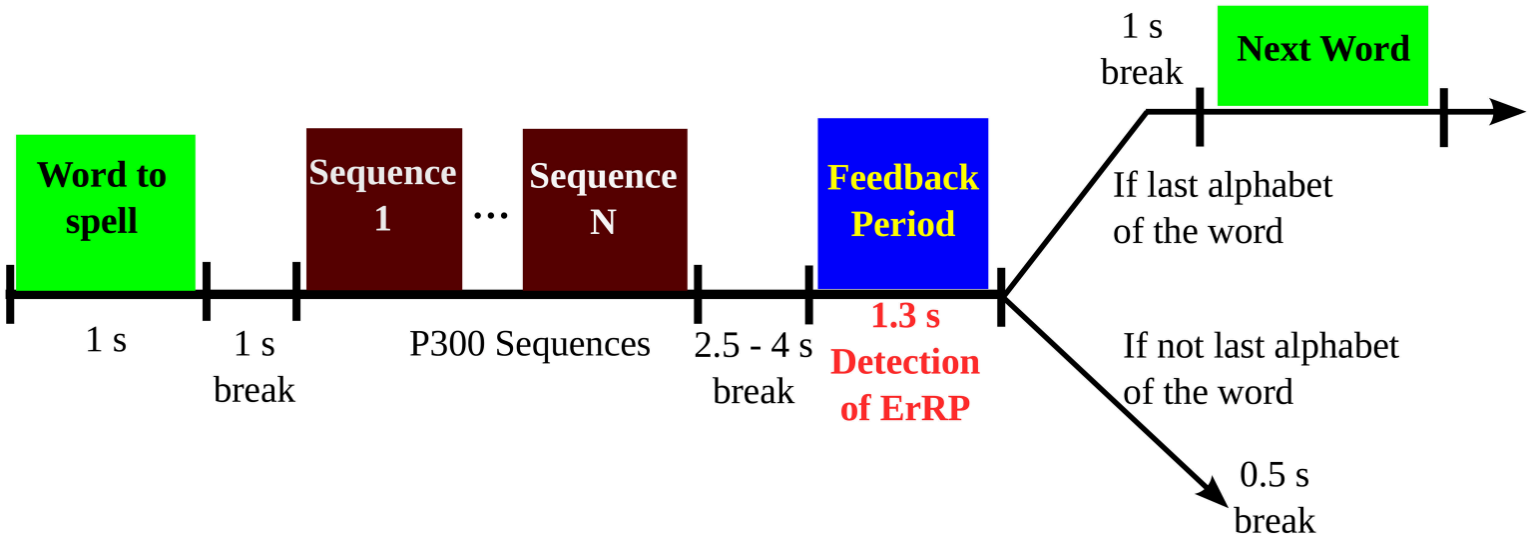
**Sequence of a trial in the P300-speller task, as given in Perrin et al. (2012)**. The feedback period is the period of interest in this study.

Two spelling conditions were used in this study, which are: (i) a fast, more error prone condition, where each item is flashed for four sequences, and (ii) a slower, less error-prone condition, where each item is flashed for eight sequences. The timing sequence of the trials are shown in Figure 2. At the start of each trial, the target spelling is displayed on top of the screen and each target alphabet within the matrix was presented by enveloping it in a green circle for 1 s. Next, sequence of stimulations are displayed with no breaks in-between. After 2.5–4 s of the last flash, the feedback is displayed in a blue square at the middle of the screen for 1.3 s. Following the last flash, the participants were instructed to keep looking at the screen with no blinking. The feedback period elicits the error response (if any) among the participants. Following the feedback session, a 0.5 s break is incorporated which marks the end of the current trial (Perrin et al., 2012). For this paper, we are working with the feedback portion (1.3 s) of each trial for each participants to detect ErrP potential in the EEG.

Each participant had undergone five separate sessions of copying the spelling of a 5-lettered word using P300. The first four sessions are made of 12 five lettered words and the fifth (last) session comprises 20 five lettered words. So, for the first four sessions there are 60 feedback periods and for the fifth session there are 100 feedback periods. Thus, a total of 16 subjects × (60 letters × 4 sessions + 100 letters) = 5,440 trials, 3,850 correct feedback (or NoError trials) and 1,590 incorrect feedbacks (or Error trials), are used as training dataset and 10 subjects × (60 letters × 4 sessions + 100 letters) = 3,400 trials, 2,411 correct feedback (or NoError trials) and 989 incorrect feedbacks (or Error trials), are used as independent testing dataset.

## 3. Methods

### 3.1. General online error detection paradigm

**Figure 3:**
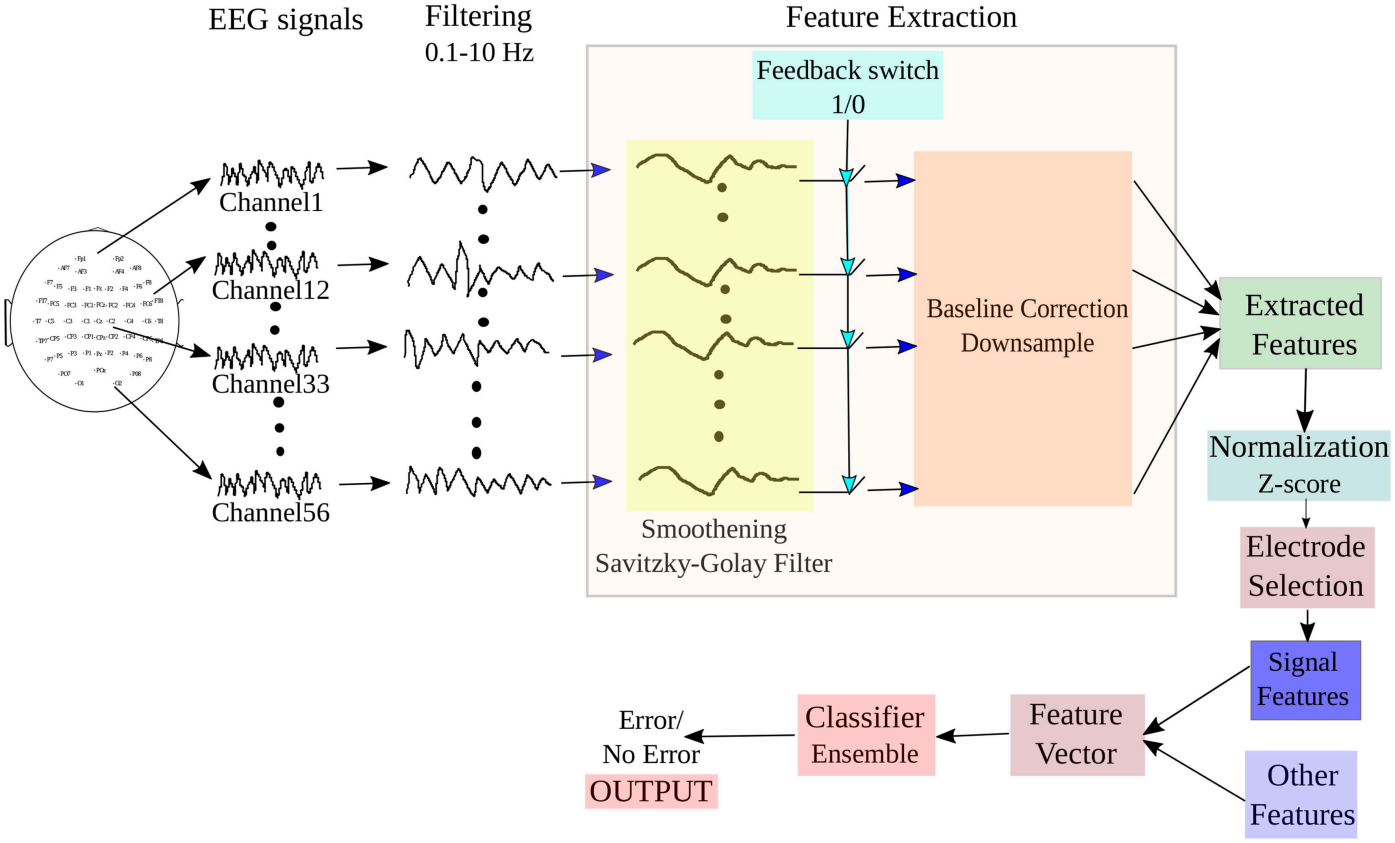
**Block diagram of the BCI system adopted for online detection of ErrP signals from the input EEG**.

The block diagram of the BCI system adopted for online ErrP detection from input EEG signals is shown in Figure 3. The system implements three main processes: (i) Pre-processing of the signal, (ii) Extraction of relevant features corresponding to the mental state from the signal, (iii) Selection of relevant electrodes and generation of feature vectors, and iii) Classification of the features to detect the intention of the participant from two given states: *Error* (or incorrect feedbacks) and *NoError* (or correct feedbacks). A switch is incorporated in the design to detect the beginning of feedback period in the trials, which is marked in the datasets. We have tested the online functionality of the BCI system on the test dataset provided in the website. To simulate a real-time condition, the EEG is continuously streamed until an onset of the feedback period is detected. On detection of the feedback period, the system extracts a pre-defined length of signal for further processing and the rest are rejected. The selected signal then undergoes filtering, feature extraction and finally are fed to a classifier to yield the required output.

### 3.2. Pre-processing

Along with the relevant EEG corresponding to the brain activity of the task performed by the participant, the signals acquired from the EEG recorder may also consist of information acquired from other brain activities (not related to the tasks). This EEG termed as *background EEG*, may be detrimental to the detection of the signature features of a particular task and thus, can be considered as noise. Other forms of noise prevalent in EEG occurs due to muscle or eye movement, line noise and other stray noise from the environment. To remove the artifacts and extract the relevant information from the signal, researchers employ different types of spatial or temporal filtering techniques (Dornhege, 2007).

**Figure 4:**
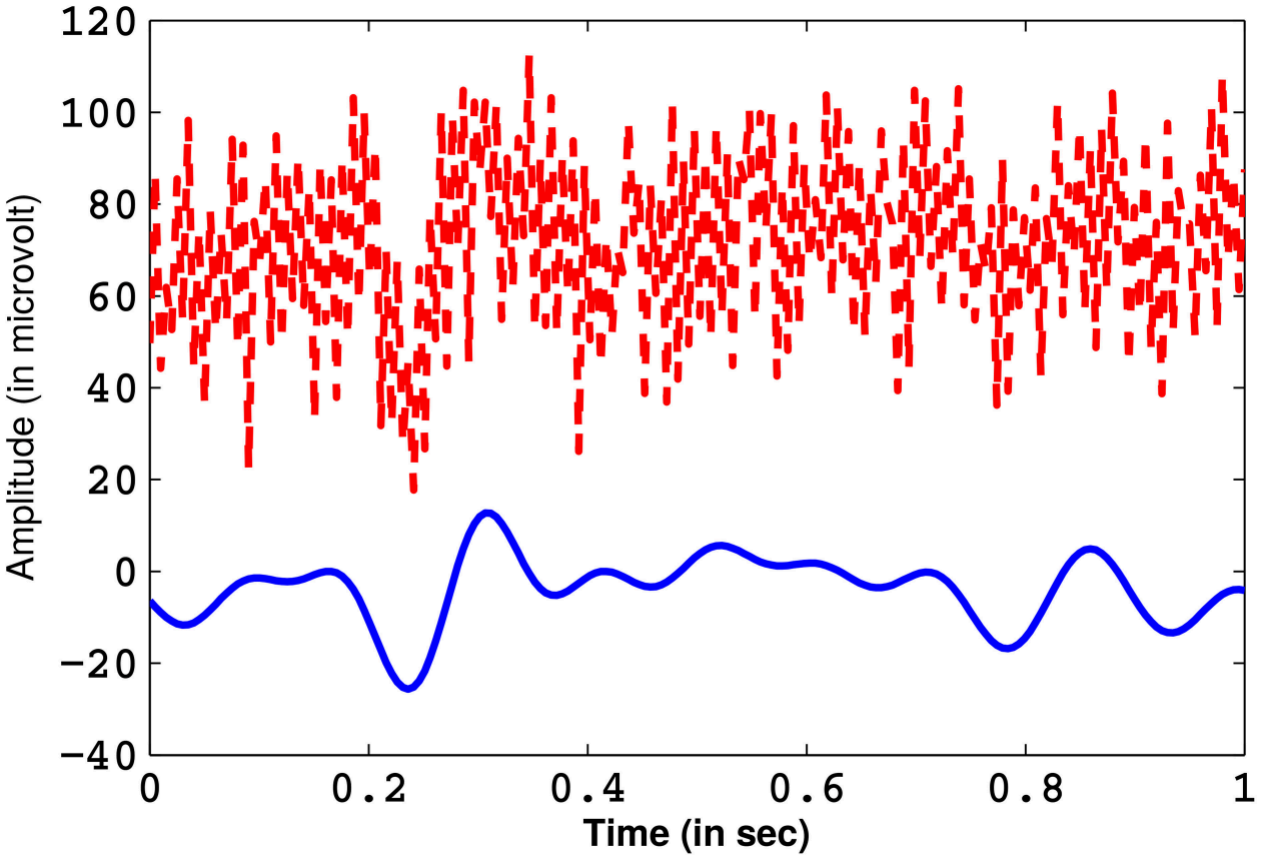
**The ErrP signal obtained from channel Cz after filtering (in blue) and its unfiltered counterpart (in red)**.

It is known from previous literature that ErrP signals are dominant in the frequency range of 0.1–10 Hz (Ferrez and Millan, 2008; Combaz et al., 2012). The incoming EEG signals (for each electrode channels) are band-pass filtered with a 0.1–10 Hz pass-band using an IIR (impulse invariant response) elliptical filter (Oppenheim et al., 1999) of order 4. The pass- and stop-band attenuation are 1 and 50 dB, respectively. IIR filters are very efficient tools of filtering of digital signals and they require less computational time when compared to other filters (Oppenheim et al., 1999). Elliptical filters are characterized by a very sharp frequency roll-off and is equi-ripple in nature, which provides good attenuation of the pass- and the stop-band ripples (Bhattacharyya et al., 2015). A comparison of a pre-processed EEG from Cz channel, containing the ErrP waveform, with its unfiltered counterpart is shown in Figure 4. Next, the EOG artifacts (if any) are removed from the EEG signals through blind source separation using independent component analysis (Jung et al., 2000). Finally, the signal that is derived is free from all form of noise.

### 3.3. Feature extraction

#### 3.3.1. Signal features using Savitzky-Golay filter

After filtering the incoming EEG signal in the required frequency range and removing other stray influences from the signal, it is furthered smoothened by using Savitzky-Golay (SG) filter (Schafer, 2011). Savitzky and Golay (1964) proposed a method of smoothening noisy data using local least-squares polynomial approximation. Moving averages (Chen and Chen, 2003) tends to flatten and widen the peaks in a spectrum, which can lead to misleading conclusions while analyzing a signal. The main idea behind the SG filter was to smoothen the data while preserving the features of the signal distribution. To meet this requirement, a linear regression of some polynomial is performed individually for each sample, followed by an evaluation of the polynomial for that sample. The key-point in this method is that the coefficients for the regression of a polynomial of a finite power is calculated only once in an early stage and then computing a convolution of the discretely sampled input data with the coefficient vector. Now, since the coefficient vectors are smaller in size than the data vector, the calculation of the convolutions are fast and straight-forward to implement. If we consider the vectors to be (*A*_−*n*_, *A*_−(*n*−1)_, …, *A*_*n*−1_, *A*_*n*_), then a smoothed sample point using SG is

(1) $$\begin{array}{r} {\left(y_{k}\right)}_{s}=\frac{\sum_{i\;=\;-n}^{n}A_{i}y_{k+i}}{\sum_{i\;=\;-n}^{n}A_{i}} \end{array}$$

**Figure 5:**
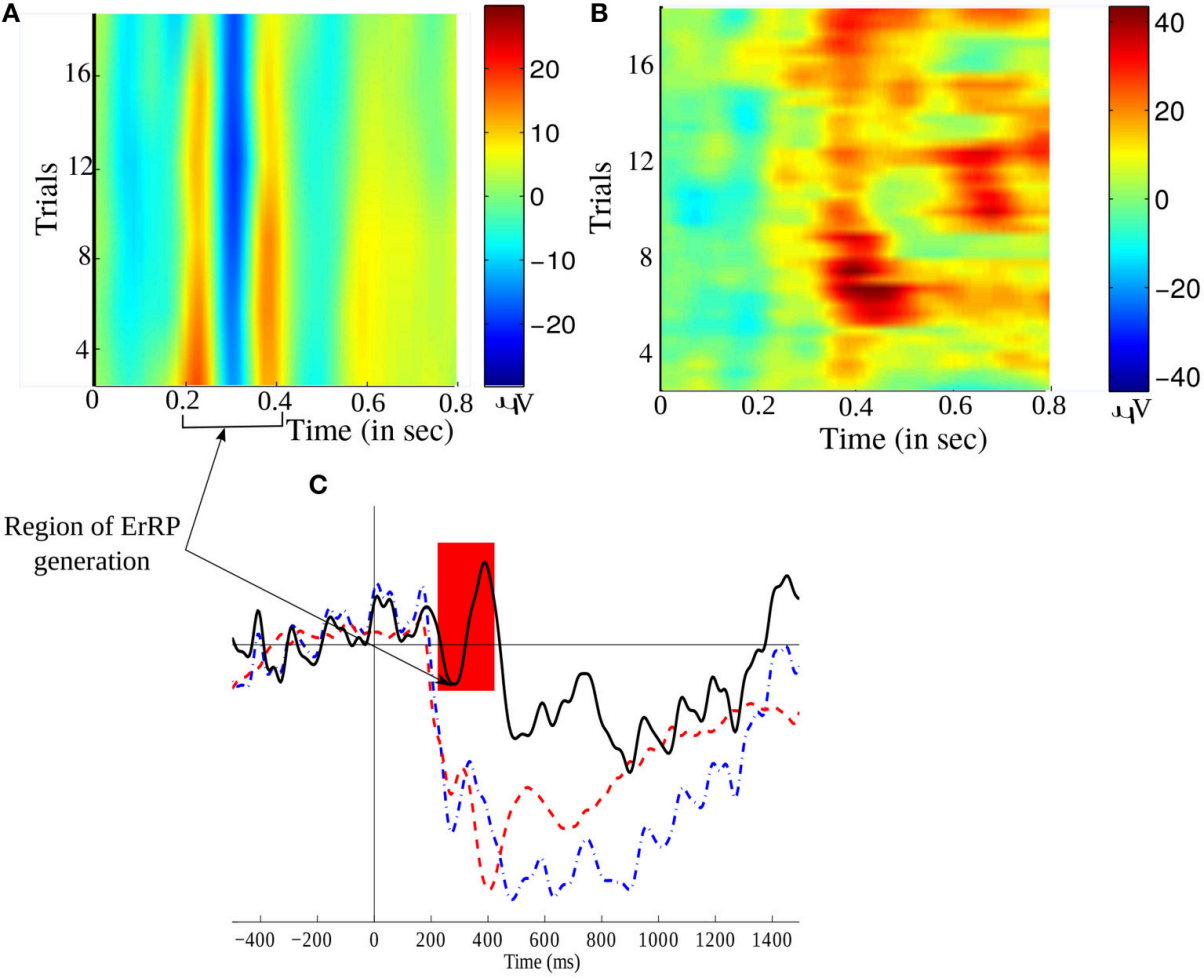
**Time-trial representation of (A)** error related EEG signal, and **(B)** No error related EEG signal, over all trials. **(C)** A comparison of the grand averaged ErrP (in black –), non-ErrP (in blue –.) and their difference signals (in red –) from channel Cz. The shaded region marks the occurrence of the typical ERN waveform.

After several experimentation, we found that the polynomial order of 3 and window size of 31 is the best to discriminate the ErrP signals (present in Error trials) from the non-ErrP ones (present in noError trials). Figure 5 illustrates the grand average of Error trials, noError trials, and their difference obtained from channel Cz. Figures 5A,B gives a time-trial representation of the smoothed EEG during error and no error feedback, respectively. The red color in the figures marks the period of high intensity of EEG signals while performing the task. Since, we are working on a feedback task, we expect the ErrP to have a postive peak at around 200ms, followed by a large negative peak at around 250 ms and again a positive deflection at around 320ms, which is evident in Figure 5C. Figure 5C shows the large difference in the waveform for error and no error condition.

Then, the signals from *t*−200 to *t*+1000 ms, where *t* coincides to the onset of the feedback period are extracted. The extracted signals from *t* ms to *t*+1000 ms are then baseline corrected by subtracting the average of the sequence *t*−200 ms to *t* ms. We perform this step to negate the effect of the background EEG from the relevant EEG. Then, we downsample the features for each electrode by a factor of 20. This reduces the features for each electrode from 200 to 8. We have included this step to reduce the computational time of the BCI system, to make it more suitable for real-time tasks and to prevent over-fitting on the training of the classifiers. The final dimensions of the signal features for each trial are 56 electrodes × 8 features = 448 features.

#### 3.3.2. Other features

Other than the signal features, we have included the following features related to the experimental tasks at hand.

1. **Mean:** It is the average of the filtered signals for each trial, following baseline correction.
2. **Variance:** It is the variance of the filtered signals for each trial, following baseline correction.
3. **Session Number:** The number of the session the current epoch exists in. It provides information of the level of training of the participant. Since, each participant underwent five sessions, thus, this feature is denoted by integer value {1, 2, 3, 4, 5}.
4. **Feedback Number:** The count of the feedback after the beginning of the current session. This feature is again denoted by integer values from {1, 2, …, 60} for the first four session and {1, 2, …, 100} for the fifth session.
5. **Alphabet Position:** The position of the letter in the current word in the current trial. In this experiment, each word is made of 5 alphabet position and thus, the features are made of integer values {1, 2, 3, 4, 5}.
6. **Word Number:** The count of the current word since the beginning of the current session. The first four sessions include 12 words and the fifth session includes 20 words.
7. **Total feedback:** It is the total number of feedbacks counted from the beginning of the first session. The features are integer values.
8. **Total Word:** It is the total number of words counted from the beginning of the first session.
9. **Sequence type:** This feature denotes whether the trial is a long or a short sequence. Long sequences are denoted by 1 and short sequences are denoted by 0.

In summary, the final size of this set of features for each trial is 9. Therefore, the total size of the feature vector is 457 features.

### 3.4. Selection of relevant electrodes

**Figure 6:**
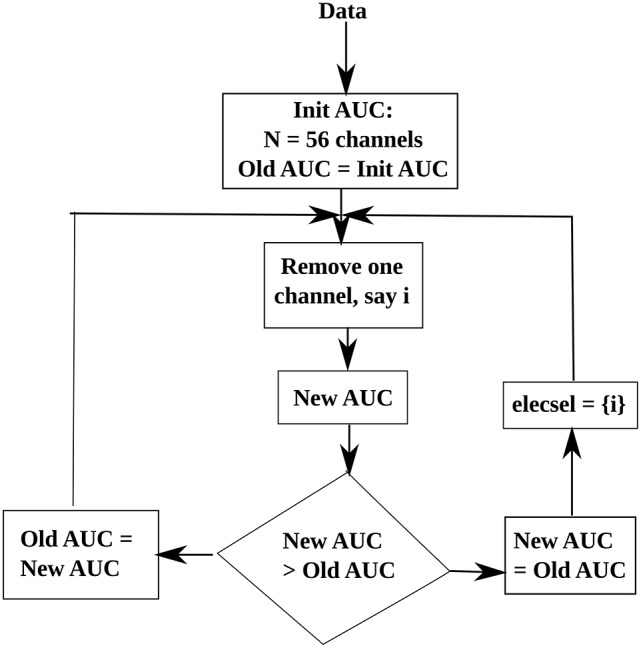
**Flowchart of the electrode selection algorithm**. Here, elecsel stands for a subset of selected electrodes (or channels).

To reduce the computational time of the decoder, while maintaining an adequate level of classifier performance, we have used a reduced set of electrodes. Thus, we have employed the backward elimination method to obtain an optimal set of electrodes for classification. Backward elimination (McCann et al., 2015), is a class of greedy algorithms, which first analyses the whole set of data and at each iteration, it removes an element to create a new smaller subset until an optimal value is reached. In our study, we aim to select an optimal subset of electrodes, thus, first we obtain the initial classification Area Under the Curve (AUC) value for 56 electrodes. Then, at each iteration, we remove an electrode and calculate a new AUC. If the new AUC-value is smaller than the old AUC, then the current electrode is added to the optimal electrode subset, and the algorithm moves toward the next step. If the current AUC is greater than the old AUC then that electrode is rejected. A flowchart is shown in Figure 6 to illustrate our electrode selection technique. Finally, the features corresponding to the selected electrodes were used to construct the feature vector, to be used as inputs to the classifier.

### 3.5. Designing the ensemble decoder

The decoder designed for this study employs an ensemble approach (Hastie et al., 2001) toward classification. The premise of our ensemble approach follows the following steps:

Let us consider a data pair (*X*_*i*_, *Y*_*i*_), (*i* = 1, …, *n*), where $X_{i}\;\epsilon \;{ℜ}^{d}$ denotes the *d*−dimensional feature vector and *Y*_*i*_ ϵ {0, 1, …, *C*−1}, where *C* is the number of classes (which in our study, is *Error* and *NoError*) and *n* is the number of training observations or samples. For a classification problem, the target function can be given by *P*[*Y* = *c*|*X* = *x*], (*c* = 1, …, *C*−1) and the function estimator is

(2) $$\begin{array}{r} ĝ\left(.\right)=h_{n}\left(\left(X_{1},Y_{1}\right),\left(X_{2},Y_{2}\right),\ldots ,\left(X_{n},Y_{n}\right)\right)\left(.\right):{ℜ}^{d}\to ℜ \end{array}$$

where, *h*_*n*_(.) is a function which estimates the features.

1. Construct training samples $\left(X_{1}^{⋆},Y_{1}^{⋆}\right)\ldots \left(X_{m}^{⋆},Y_{m}^{⋆}\right)$ for each individual learner by randomly selecting *m* samples with replacement from the original samples (*X*_1_, *Y*_1_)…(*X*_*n*_, *Y*_*n*_). The random selection of the samples is done using *k*-fold technique.
2. Compute the function estimator ĝ^⋆^(.) similar to (2), for each learner. Here, we use the posterior probability of each learner as estimator of the function, which is given as
  (3) $$\begin{array}{r} {ĝ}^{⋆}\left(.\right)=\hat{P}\left[Y^{⋆}=c|X^{⋆}=.\right] \end{array}$$
3. Repeat Step 1 and 2 *M* times, where *M* is an integer value defined by the user, producing ĝ^⋆, *m*^(.), where *m* = 1, 2, ….*M*. The final estimation ĝ_*ens*_(.) of the ensemble for *P*[*Y* = *c*|*X* =.] is the average of all the probabilities obtained from each individual learner, which is
  (4) $$\begin{array}{r} {ĝ}_{ens}\left(.\right)=\frac{1}{LM}\overset{M}{\underset{m\;=\;1}{\sum }}{ĝ}^{⋆,m}\left(.\right) \end{array}$$
  where, *L* is the number of weak learners.

**Figure 7:**
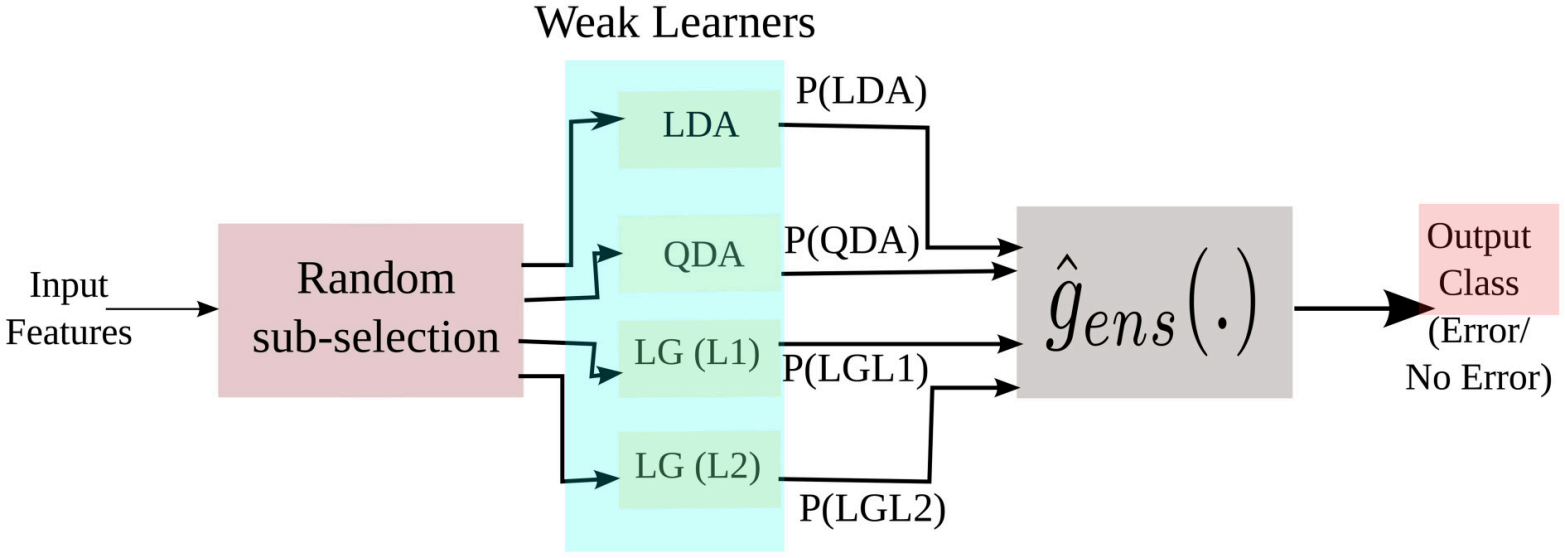
**A simplified representation of our ensemble approach during training**. P(.) are the posterior probabilities of the weak learners.

In this study, we have used Linear Discriminant Analysis (LDA), Quadratic Discriminant Analysis (QDA) with regularization value of 0.07, Logistic Regression (LG) with L1 and L2 regularization (Hastie et al., 2001; Alpaydin, 2004) with a regularization value of 0.15. LDA aims at separating the data representing the different classes by constructing a linear hyperplane, by assuming normal distribution of the data with equal covariance matrix for all classes. Here, the class of an observation depends on which side of the hyperplane the feature vector falls. The separating hyperplane is a projection that maximizes the distance between two class means and minimizes the inter-class variance. QDA is similar to LDA in most respect except it assumes that the covariance matrix are different for each class and it has more parameters to estimate. Logistic regression is a probabilistic type of classifier which predicts the outcome (or classes) of one or more features based on a logistic function, $g\left(n\right)=\frac{1}{1+e^{-n}}$ where *n* is the linear combination of the input features. This classifier measures the relationship between the classes and the features by using the probability scores as the predicted value of the classes. In short, it predicts the probability of the class to be positive (Hastie et al., 2001; Alpaydin, 2004). In L1 regularized LG [LG(L1)] the number of irrelevant features grow logarithmically whereas in L2 regularized LG [LG(L2)] the number of relevant features grow linearly (Ng, 2004). All the three classifiers are easy to implement and computationally fast. A simplified block diagram of the implementation of the three classifiers in the ensemble framework is shown in Figure 7.

### 3.6. Design for online processing

As mentioned earlier, in this study, we are concerned only with the detection of ErrP signals and in general, occurrence of error, from the feedback period of the experiment. Each dataset contains information of the onset of feedback period for each trial. The time instances marked as “1” corresponds to the beginning of the feedback period while “0” marked the non-feedback periods. As the signals were already provided earlier in the competition, in this study we have simulated an online processing environment and thus, our test environment is “*pseudo-online*” in nature. During online processing of the EEG signal, we introduced a feedback switch in our system, which behaved as an ON/OFF switch with “1” being the “ON” state. If the switch observed an ON at time instance *t* ms, the BCI system would extract the EEG signals from *t*–200 ms to *t*+1000 ms for analysis. Then, the signal block would be filtered and smoothened using SG filter. For smoothening to occur in an online scenario, 100 ms of EEG from the previous trial are included in the current block of EEG and then SG filter is applied. Following the smoothening, the signal would be baseline corrected and down-sampled. It must be noted during online processing, we only work with electrode channels selected during the training (offline) process. The features are then fed to the trained classifier to generate the necessary output.

### 3.7. Evaluation metrics

To evaluate the performance of the BCI system, we have employed three quantitative measures. They are: (i) Classification Accuracy (Alpaydin, 2004), (ii) F1-score (Goutte and Gaussier, 2005), and (iii) Area Under the Curve (AUC) (Hanley and McNeil, 1982). The metrics can be summarized as follows:

1. **Classification Accuracy (or Acc):** It is the measure of how correctly a classifier can predict a class (Alpaydin, 2004).
2. **F1-score (F1):** The F1-score of the classifier is the harmonic mean of precision and recall (Goutte and Gaussier, 2005), and is given as
  (5) $$\begin{array}{r} F1-score=\frac{2\times precision\times recall}{precision+recall} \end{array}$$
  For a problem with uneven class distribution, such as our problem, F1-score is more useful than accuracy because it takes into account both false positives and false negatives.
3. **Area Under the Curve (AUC):** AUC is derived from the Receiver Operating Characteristic (ROC) (Fatourechi et al., 2008) curve of the classifier performance. ROC curve is a plot of the classification result of the most positive classification to the most negative classification and perfect classification is denoted by a point (0,1) in the upper left corner. The random guess line in the curve is the line joining (0,0) and (1,1) and contains the point (0.5,0.5). This line divides the ROC space in two portions. Points in the upper portion of the random guess line indicate good prediction and the points below the line indicate poor prediction. The resultant area under the curve is widely used as a classification metric.
4. **Computation Time (CT):** The time required by the trained decoder to produce an output for a single trial. This metric is used during the simulated online testing of the decoder. It is given in microseconds.

### 3.8. Statistical validation using friedman test

Friedman test (Chen et al., 2009), compares the relative performance of proposed ensemble classifier with the eight standard classifiers. The null hypothesis here, states that all the algorithms are equivalent, so their ranks *r*_*j*_ should be equal. The Friedman statistic, is distributed accordingly to $\chi_{F}^{2}$ with *k*−1 degrees of freedom, which is calculated as

(6) $$\begin{array}{r} \chi_{F}^{2}=\frac{12N}{K\left(K+1\right)}\left[\underset{j}{\sum }R_{j}^{2}-\frac{K{\left(K+1\right)}^{2}}{4}\right] \end{array}$$

## 4. Results

This section provides the results on the performance of the BCI system for the proposed error potential detection method. The first sub-section comprises the electrode selection and training results of the ensemble classifier followed by a statistical validation of our ensemble classifier with standard classification algorithms, which are, LDA, QDA, Logistic Regression and Support Vector Machines (SVM), and standard ensemble techniques like Adaboost (Ada) classifier, Bagging (Bag) classifier, and Gradient Boosting Machine (GBM) classifier. The following section provides the result during online testing of the decoder and compares the result with other competitive algorithms. The analysis of the work has been done on a python environment in an ubuntu 15.04 based computer system with 8GB ram and AMD A10 1.89 GHz processor.

### 4.1. Offline training analysis

**Figure 8:**
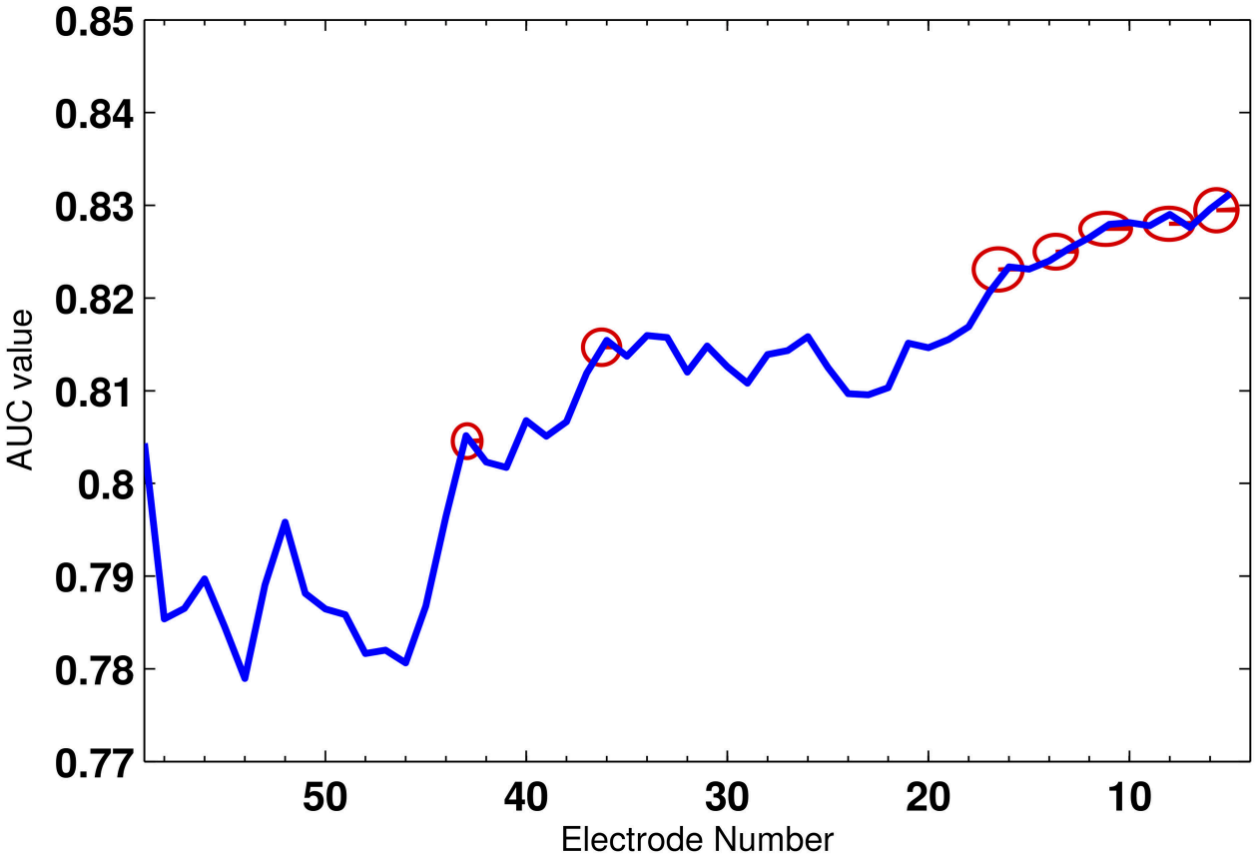
**The average AUC value across 56 electrodes**. The red circles mark the electrodes which were rejected.

First, we select the optimal set of electrodes using the algorithm explained in Section 3.4 on the training set. The features of all the subsets of electrodes are first pooled together and then *k*-fold cross validation (Alpaydin, 2004) is employed to reduce the variance in the performance of the decoder. Here, we have selected *k* as 10. The cross validation technique divides the training set to two different subsets: one to train the classifier and the other to validate the feature selection and classification performance. Figure 8 shows the average AUC values of all iterations during the electrode selection for the validation subset. As observed from the figure, the electrodes, whose removal had increased were the AUC rejected from the optimal electrode subset. The red circles gives an example of few electrodes which were removed. Finally, a total of 35 electrodes were selected to construct the feature vector and used for classification.

The average validation results, i.e., Accuracy, F1-score and AUC, for the selected subset of electrodes is shown in Table 1. The table shows that the Accuracy and AUC training results obtained are above 80%, which is commendable considering the fact that it is a cross-subject evaluation for a transferable decoder. In our study, the F1-score obtained is more than 94% which suggests a good performance both in terms of precision and recall Goutte and Gaussier (2005).

**Table 1:** **Performance validation of the ensemble classifier with its individual components using 10-fold cross validation**.

	**Acc (in %)**	**F1 (in %)**	**AUC (in %)**
LDA	74.50 ± 1.32	83.40 ± 1.12	73.42 ± 1.86
QDA	73.47 ± 1.28	82.31 ± 1.11	73.88 ± 1.61
LGL1	74.21 ± 1.66	83.75 ± 1.20	73.49 ± 1.94
LGL2	70.77 ± 0.89	82.88 ± 0.61	54.55 ± 2.75
SVM	73.68 ± 1.46	82.67 ± 0.83	73.45 ± 1.56
Bag	73.42 ± 1.32	83.26 ± 0.83	74.34 ± 0.98
Ada	72.00 ± 0.73	82.04 ± 0.61	71.57 ± 2.60
GBM	72.92 ± 1.12	82.79 ± 0.77	69.47 ± 0.58
Ensemble	81.42 ± 0.98	94.32 ± 0.67	83.13 ± 1.71
(Proposed)			

*The values are the average across 10 runs*.

Table 1 also compares the ensemble result with standard classifier result and it can be seen that the ensemble method increases the performance of the decoder by more than 6% from the “best” standard classifier. The ranking of each performance metrics for each individual classifiers differ. For instance, as seen in Table 1, LDA has the second-best accuracy but the third-best F1-score, while LG(L1) has the third-best accuracy, second-best F1-score, and fourth-best AUC. Such kind of results may confuse the operator in selection of the right classifier for the task at hand. In this regard, the ensemble method provides a stability as they yield a uniform performance for all metrics.

### 4.2. Online ErrP detection test results

After selecting the optimal electrode subset and training the classifier, we test the performance of the decoder on an independent new dataset of 10 subjects, which is also the test dataset provided in the competition (BCI Challenge, 2015). Here, we have simulated a real-time acquisition and processing condition on the test dataset. A feedback switch is included in the system to identify the manifestation of the feedback period. The EEG data is streamed in a continuous manner until the switch detects an onset of the feedback. On detection, the system creates a data block from –200 to 1000 ms from the beginning of the feedback period which are then used for processing. The accuracy, F1-score, and AUC results of the test dataset in this pseudo-online condition is shown in Table 2. Here, we have shown the results of the proposed ensemble classifier (trained by the selected electrodes) and again compared the results with the standard classifiers. Similar to the training results, we have also compared the performance of the ensemble with its individual components. The results follow a same trend as the one obtained during 10-fold cross-validation phase with the proposed ensemble classifier performing better than the rest.

**Table 2:** **Online performance of the ensemble classifier for selected and all subjects and comparison with its individual components**.

**Subjects**	**Acc**	**F1**	**AUC**	**CT**
Ensemble	73.97	83.53	73.18	3,425
(Proposed)				
LDA	73.32	82.53	72.93	1,134
QDA	69.26	79.82	68.48	1,247
LG(L1)	72.73	82.89	70.10	824
LG(L2)	70.91	82.98	59.14	576
SVM	72.22	82.76	71.45	1,456
Bag	73.00	82.30	72.44	4,823
Ada	71.02	81.62	66.26	5,188
GBM	71.29	82.04	66.67	5,540

For the ensemble classifier, the difference between the validation results and the test results, i.e., the test accuracy, F1-score, and AUC differ by 7.45, 10.79, and 9.95% to its cross-validated counterpart. Next, we study the individual classification performance for each subject and the results are presented in Table 3. It is noted that all few subjects, such as 03, 05, 19, and 25 have poor individual performance. Thus, the sizeable difference between the cross-validated results and the test results can be attritbuted to these subjects. Subject 10 has the best results of 88.53% accuracy, 93.70% F1-score, and 78.06% AUC, whereas Subject 5 has the worst performance. It is noted that 6 of 10 subjects have performance more than 77% which shows the efficiency of our transferable BCI decoder.

**Table 3:** **Online test results of the ensemble classifier for individual subjects**.

**Test ID**	**Acc**	**F1**	**AUC**
01	81.76	89.42	73.37
03	61.17	66.33	75.59
04	87.35	92.49	88.77
05	42.35	59.17	62.99
08	77.06	85.71	80.71
09	82.06	89.68	77.45
10	88.53	93.70	78.06
15	87.94	93.38	77.31
19	68.82	78.80	72.67
25	62.35	72.76	72.52

On the one hand, the results shown by our proposed decoder is comparable to the standard classifiers. Further, we have also shown a better computational cost through our generic tranferable decoder by a significant amount as compared to standard ensemble techniques, which is highly beneficial for a real-time detection problem. In addition, the emsemble method presented stable performance for all the performance metrics also in the case of on-line ErrP detection for unknown dataset with generic transferable decoder.

### 4.3. Statistical validation

We have considered the performance of the classifiers during cross-validation for statistical evaluation. Here, we have considered *K* to be the number of classification algorithms in competition and *N* is the number of performance metric used during cross-validation. The individual and average rankings while validating the classifiers are provided in Table 4 and while testing the classifiers are provided in Table 5.

**Table 4:** **Ranking table during validation of the classifier for 11 subjects**.

**Classifiers**	***Rank***			**Average Rank**
	**Acc**	**F1**	**AUC**	***R*****_*j*_**
LDA	2	3	6	3.67
QDA	4	8	3	5
LG(L1)	3	2	4	3
LG(L2)	8	5	9	7.33
SVM	6	6	5	5.67
Bag	5	4	2	3.67
Ada	9	9	8	5.67
GBM	7	7	7	7
Ensemble	1	1	1	1

**Table 5:** **Ranking table during online testing for 10 independent subjects**.

**Classifiers**	**Rank**				**Average Rank**
	**Acc**	**F1**	**AUC**	**CT**	***R*****_*j*_**
Ensemble	1	1	1	6	2.25
LDA	2	5	2	3	3
QDA	9	9	6	4	7
LG(L1)	4	3	5	2	3.5
LG(L2)	8	2	9	1	5
SVM	5	4	4	5	4.5
Bag	3	6	3	7	4.75
Ada	7	8	8	8	7.75
GBM	6	7	7	7.25	

From Table 4 and Equation (6), $\chi_{F}^{2}$ is calculated to be 1.5858 which is >1.344 (the standard statistics value). Thus, we can conclude that for (*K*−1=9-1=) 8° of freedom and in 99.5% confidence that the null hypothesis is wrong and hence, the classifiers are not equivalent rather they are ranked according to *R*_*j*_. Similarly, From Table 5 $\chi_{F}^{2}$ is calculated to be 16.3897 is >1.344 and thus, the classfiers are ranked according to *R*_*j*_. Therefore, we have statistically validated that our ensemble classifier is better than its individual components and this ranking information itself is useful to know which classification algorithms are effective specifically for the error potential detection.

## 5. Discussion

In this work, we have designed an online transferable EEG-BCI system which detects the occurrence of error during a discrete target selection task from the recorded EEG signals from independent dataset with no training. The error is detected during the feedback period of a P300 copy-spelling task performed by the participant, when a particular feedback is incorrect. From literature Perrin et al. (2012) it is known that if the feedback is incorrect, an ErrP signal would be found in the EEG of the participant. A major component of our BCI system is the detection of specific ErrP features from the EEG features from the feedback period of the tasks. The dataset for this experiment is provided as a competition file by BCI Challenge (2015) for 26 subjects, where 16 subjects are used to train the decoder and the testing is performed on the remaining 10 subjects. It must be noted that the dataset used in the competition are similar to the one used in Perrin et al. (2012). Researchers in Perrin et al. (2012) developed an online subject-specific error detector and attained an average accuracy of 76%. In our paper, we have aimed to develop an online subject-independent transferable BCI system to detect error, in the form of ErrP, in a discrete target selection task and have produced an average accuracy of 81.42% on the training set and 73.97% on the testing set. Thus, even for a system which detects the occurence of error across different subject, we have attained a result comparable to the original paper.

We prepared a feature vector made of signal features such as the smoothed sample values using savitzky golay filter, the mean and variance of the trials, and features related to the experimental conditions, so that the classifier could learn from the changing environmental states (in this case, change of session/feedback, or a new word or alphabet position) and aid in boosting the performance. Further, we aimed at fusing the learning of different classifiers to create an ensemble output. This step was taken to avoid over-fitting among the individual classifiers and help in improving the performance of the BCI system. Further, we have selected a subset of optimal electrodes whose features are to be used for classifcation using a backward elimination algorithm.

We have also test the BCI error detection transferable decoder to be online compatible. Since, the datasets along with their corresponding classes were already provided in the competition website, we have tested the online implementation of the system in a simulated manner. We streamed the test data continuously for each participants and on detection of the feedback period, the system would extract a block of EEG data, process it and produce a result: *Error* or *NoError*.

In the competition, the participants used a separate set of features to boost the performance of the decoder, which can be termed as “leakage features.” Because of the design of the error detection system (see Perrin et al., 2012), it is possible from session 5 to determine whether a particular trial has error or not, by analyzing the time between feedback events. From this information, a feature vector can be constructed by assigning a value of 1 if the online detector (as designed in Perrin et al., 2012) has detected an error and 0 if it has not detected an error. The results (Tables 1, 2) in this paper, discussed in the previous sections, are without the “leakage features.” To bring some parity with the common trend of the competition, we have included the “leakage features” to our already prepared dataset and re-tested it on the test dataset. The results, thus, obtained using our proposed algorithm and the combination of the original features with the “leakage features,” is shown in Table 6. The performance evaluation of the competition was based on the AUC value, and thus we have shown only the AUC results in this table. Table 6 compares the AUC of the ensemble while using only “signal features,” “signal + other features,” and “signal+other+leakage” features. The AUC of features including the leakage ones have higher than the other cases. It would seem that the leakage features boosts the AUC values. The table also suggests the competitiveness of our proposed algorithm with standard classifiers and ensemble techniques (Hastie et al., 2001). GBM and Adaboost are considered to be good ensemble classifiers (Hastie et al., 2001) and our proposed algorithm has proved to be comparable to both of them. The performance by our proposed method (83.12%) is on the top three results in the BCI competition, if we locate our performance value in the final BCI competition result (BCI Challenge, 2015). The best score was 87.2% but it takes 70 min (training and prediction) on a 64 GB RAM computer to generate the results for all the subject. On the other hand, our approach takes around 16 min to generate the results from all subjects (31 s for each trial) on 8 GB RAM computer. Thus, considering a trade-off between speed and performance, our approach is practical in its implementation for real-time problems.

**Table 6:** **Comparison of AUC with standard competitor algorithms using leakage features**.

**Classifier**	***Signal***	***Signal + Other***	***Signal + Other + leakage***
Ensemble	71.31	73.18	83.12
(Proposed)			
LDA	65.42	72.93	82.56
QDA	60.67	68.48	74.33
LG(L1)	60.50	70.10	82.34
LG(L2)	47.62	59.14	77.57
SVM	65.78	71.45	82.40
Bag	63.25	72.44	72.56
Ada	55.34	66.26	76.53
GBM	59.25	66.67	76.53

As for online decoding, its performance with this dataset is reported in Perrin et al. (2012). Its performance was 63% sensitivity and 88% specificity, and it allowed subject-specific identification, and it was not a generic transferable decoder as in the case of this paper. Through our decoder, we have designed a zero-training online BCI system by removing the requirement to re-calibrate the system for every new subject.

Even though our proposed features has a lower performance than the one using leakage features, it still provides an accuracy of more than 73.97%, which is commendable for a cross-subject transferable decoder, as EEG of each subjects or sessions are different from each other. As noted from the results, inclusion of leakage features tends to improve the global metrics by optimizing the dynamics of the prediction but it will have little influence on single-trial predictions, which is one of the requirements for real-time tasks. For general BCI-based target selection applications, features without the leaked information are most suitable as then the problem is more of a single-trial prediction problem. The incorporation of the ErrP detection system along with other signal modalities like ERD/ERS, SSVEP, and P300 (as in this work) would be possible as in the pure “ErrP” result (without leakage feature) and make it closed-loop in nature to correct the erroneous selection. The usage of the “leakage feature” in this section is solely for the purpose of parity with the competition trend.

As we have tested the system on a pseudo-online environment, future works would involve testing the system on real world tasks and adapting the parameters accordingly. Also, there are scopes to improve the decoder performance from this work, especially for the computational cost aspect in the ensemble decoder.

## 6. Conclusion

The work presented in this paper describes the design of an online transferable BCI system tasked with detecting error from a discrete target selection task using EEG signals. The decoder designed for the system is ensemble and generic in nature and is designed to detect error for new participants without the requirement of a training session. The results obtained from our ensemble approach has proved to be competitive with other ensemble techniques. With some modifications in the parameters, the principle of the cross-subject cross-sessions system developed in this paper may be applied in a hybrid manner together with other BCI paradigms other than the one based on ErrP. Incorporating online detection of error with other BCI tasks has great potential in future neuro-prosthetic (Li et al., 2014), rehabilitative, and robotic control applications.

## Author contributions

SB: Algorithms, Data processing, publication writing. AK: supervision of data processing and publication review. DT: supervision of data processing and publication review. MH: Supervision of data processing and publication drafting, publication review.

### Conflict of interest statement

The authors declare that the research was conducted in the absence of any commercial or financial relationships that could be construed as a potential conflict of interest.
